# Identification of Tamoxifen-Resistant Breast Cancer Cell Lines and Drug Response Signature

**DOI:** 10.3389/fmolb.2020.564005

**Published:** 2020-12-04

**Authors:** Qingzhou Guan, Xuekun Song, Zhenzhen Zhang, Yizhi Zhang, Yating Chen, Jing Li

**Affiliations:** ^1^Co-construction Collaborative Innovation Center for Chinese Medicine and Respiratory Diseases by Henan & Education Ministry of P.R. China, Academy of Chinese Medical Sciences, Henan University of Chinese Medicine, Zhengzhou, China; ^2^College of Information Technology, Henan University of Chinese Medicine, Zhengzhou, China; ^3^Department of Bioinformatics, Key Laboratory of Ministry of Education for Gastrointestinal Cancer, School of Basic Medical Sciences, Fujian Medical University, Fuzhou, China

**Keywords:** breast cancer, tamoxifen, cell line, resistant, sensitive

## Abstract

Breast cancer cell lines are frequently used to elucidate the molecular mechanisms of the disease. However, a large proportion of cell lines are affected by problems such as mislabeling and cross-contamination. Therefore, it is of great clinical significance to select optimal breast cancer cell lines models. Using tamoxifen survival-related genes from breast cancer tissues as the gold standard, we selected the optimal cell line model to represent the characteristics of clinical tissue samples. Moreover, using relative expression orderings of gene pairs, we developed a gene pair signature that could predict tamoxifen therapy outcomes. Based on 235 consistently identified survival-related genes from datasets GSE17705 and GSE6532, we found that only the differentially expressed genes (DEGs) from the cell line dataset GSE26459 were significantly reproducible in tissue samples (binomial test, *p* = 2.13E-07). Finally, using the consistent DEGs from cell line dataset GSE26459 and tissue samples, we used the transcriptional qualitative feature to develop a two-gene pair (*TOP2A*, *SLC7A5*; *NMU*, *PDSS1*) for predicting clinical tamoxifen resistance in the training data (logrank *p* = 1.98E-07); this signature was verified using an independent dataset (logrank *p* = 0.009909). Our results indicate that the cell line model from dataset GSE26459 provides a good representation of the characteristics of clinical tissue samples; thus, it will be a good choice for the selection of drug-resistant and drug-sensitive breast cancer cell lines in the future. Moreover, our signature could predict tamoxifen treatment outcomes in breast cancer patients.

## Introduction

The overall recurrence rate of estrogen receptor positive (ER+) early breast cancer can be reduced by adjuvant treatment with tamoxifen. However, approximately 30–40% of ER + breast cancer patients receiving adjuvant tamoxifen therapy still would relapse or progress to deadly advanced metastatic stages within 15 years follow-up; this is largely attributed to tamoxifen resistance (Ye et al., 2019). Therefore, it is of great clinical significance to identify the efficacy of tamoxifen in ER + breast cancer patients. Cell lines are a common modeling tool in cancer research (Domcke et al., 2013); they can help us to better understand the biological processes and molecular mechanisms of cancer and aid in the development of anticancer drugs (Kong and Yamori, 2012; Knudsen et al., 2014). However, whether cell line models could adequately reflect the characteristics of clinical tissue samples is controversial (American Type Culture Collection Standards Development Organization Workgroup ASN-0002, 2010; Liedtke et al., 2010; Bayer et al., 2013; Capes-Davis et al., 2019; Wass et al., 2019). It is well known that tumor cell lines might lose some of their tumor-related characteristics owing to the culture environment (Masters, 2000). Cross-contamination (International Cell Line Authentication Committee, 2014) and misidentification (American Type Culture Collection Standards Development Organization Workgroup ASN-0002, 2010) of cell lines exacerbates such issues. Moreover, there is no unified gold standard for the identification of drug-resistant cell lines, which also results in some cell lines poorly reflecting the characteristics of clinical tissue samples (Liedtke et al., 2010). Thus, it is of great value to find resistant/sensitive cell line models that are more representative of clinical tissue samples.

Considering tamoxifen survival-related genes from breast cancer tissue samples as the gold standard, we screened for the optimal cell line model. In the survival-related analysis of tissue samples, we assumed that genes that were positively (negatively) correlated with survival risk in tissue samples were comparable with genes that are upregulated (downregulated) in resistant compared with sensitive cell lines. In this study, through evaluating the consistency of prognosis-related genes in tissue samples from patients undergoing tamoxifen treatment with drug-resistance genes in cell lines, we selected the optimal cell line model to represent the characteristics of clinical tissue samples; the consistent genes between tissues and cell lines were identified as clinical drug-resistance-related genes.

Moreover, the relative expression orderings (REOs) of gene pairs within individual samples, also called qualitative transcriptional characteristics, are robust against experimental batch effects and can be directly applied to samples at an individual level (Eddy et al., 2010; Guan et al., 2019). The robustness property of the qualitative transcriptional characteristics enables integration of multiple datasets from different sources to develop disease signatures or classifiers, which improves the probability of finding robust signatures (Xu et al., 2008; Guan et al., 2019). Thus, based on qualitative transcriptional characteristics and the clinical drug-resistance-related genes that we identified, we developed a tamoxifen-resistance signature for ER + breast cancer and verified it in independent data.

## Materials and Methods

### Data and Preprocessing

Breast cancer gene expression data and corresponding clinical information were downloaded from the GEO database (Gene Expression Omnibus, http://www.ncbi.nlm.nih.gov/geo/). Relapse-free survival (RFS) time was defined as the interval between the first day of surgery and the date of death from any cause or of recurrence (local and/or distant) (Punt et al., 2007; Merok et al., 2013). Breast cancer tissue samples from ER+ patients who had received post-operative tamoxifen treatment were selected from the seven datasets, as described in Table 1. Nine gene expression datasets for breast cancer tamoxifen-resistant/sensitive cell lines were also downloaded from the GEO database, as shown in Table 1.

**TABLE 1:** Data used in this study.

Tissue					

GEO Acc	Platform	ER+ Sample	Endpoint		
GSE17705	Affymetrix GPL96	298	RFS		
GSE6532	Affymetrix GPL96	176	RFS		
GSE12093	Affymetrix GPL96	136	RFS		
GSE4922	Affymetrix GPL96	66	RFS		
GSE2990	Affymetrix GPL96	54	RFS		
GSE42568	Affymetrix GPL570	67	RFS		
GSE9195	Affymetrix GPL570	77	RFS		

**Cell line**					

**GEO Acc**	**Platform**	**Sensitive**	**Resistant**	**Sample (R vs S)**	**Method**

GSE27473	Affymetrix GPL570	MCF7	MCF7 silenced ER	3:3	RNA silencing
GSE12708	Affymetrix GPL96	SUM44	SUM44/LCCTam	3:3	Drug pressure
GSE26459	Affymetrix GPL570	B7	G11OH-T	3:3	MCF7 subclones
GSE8562	Affymetrix GPL96	MCF7	MCF7/XBP1	3:3	XBP1 overexpression
GSE14986	Affymetrix GPL570	MCF7	T8, T17, T29, T52	4:3	Drug pressure
GSE21618	Affymetrix GPL570	WT	tamR	20:11	Drug pressure
GSE67916	Affymetrix GPL570	MCF7	MCF-7/TAMR	10:8	Drug pressure
^#^GSE118713	Illumina GPL16791	MCF7	MCF-7/TAMR	3:3	Drug pressure
^#^GSE125738	HiSeq GPL20795	T47D	T47D-TR	3:3	Drug pressure

*RFS: relapse-free survival; ER: estrogen receptor. Sample (R vs S) denotes the number of the resistant and sensitive cell line sample from the corresponding dataset; Method denotes the production process for tamoxifen-resistant breast cancer cell lines. ^#^High-throughput sequencing data.*

For the array data measured by Affymetrix platform, raw mRNA expression data (.CEL files) were downloaded, and the Robust Multi-array Average algorithm was used for normalization with Affy package in R software (Bolstad et al., 2003; Irizarry et al., 2003). For sequence-based data, the processed data were directly downloaded.

### Identification of Survival-related Genes in Tissue

The Cox proportional hazard model was used to study the relationships between gene expression levels and survival (Kreike et al., 2010). For the coefficient β obtained from the Cox model, if β > 0 for a certain gene, this gene was considered to be positively correlated with survival risk and was comparable with the upregulated gene between resistant and sensitive cell lines. Similarly, if β < 0, the gene was comparable with the downregulated gene between resistant and sensitive cell lines.

### Identification of Differentially Expressed Genes (DEGs) in Cell Lines

In this study, the SAM (significance analysis of microarrays) algorithm (Tusher et al., 2001) was used to identify DEGs between resistant and sensitive cell lines.

### Consistency Evaluation Between Tissues and Cell Lines

In this study, we hypothesized that genes positively (negatively) associated with survival in tissues corresponded to those genes upregulated (downregulated) between resistant and sensitive cell lines.

The consistency ratio, which is the number of overlapping and consistent DEGs/number of overlapping DEGs, was used to evaluate the similarity between tissues and cell lines. The significance was evaluated by the binomial distribution test as follows:

$$p=1-\sum_{i=0}^{k-1}\left(\begin{array}{c} n\\ i \end{array}\right)\,0.5^{i}\,{\left(1-0.5\right)}^{n-i}$$

where *n* denotes the number of overlapping DEGs between tissue and cell line, and *k* denotes the number of those overlapping DEGs with the same dysregulation direction.

Then, the *p*-values were adjusted using the Benjamini-Hochberg method (Benjamini and Hochberg, 1995).

### KEGG Pathway Enrichment

The hypergeometric distribution model was used to determine the significance of KEGG (Kanehisa and Goto, 2000) (Kyoto Encyclopedia of Genes and Genomes) pathways enriched with the genes of interest using the following statistical model:

$$p=1-\sum_{\text{i}=0}^{\text{k}-1}\frac{\left(\begin{array}{c} \text{m}\\ \text{i} \end{array}\right)\,\left(\begin{array}{c} \text{N}-\text{m}\\ \text{n}-\text{i} \end{array}\right)}{\left(\begin{array}{c} \text{N}\\ \text{n} \end{array}\right)}$$

where *N* denotes the number of background genes, *n* denotes the number of genes of interest, *m* denotes the number of genes in a given pathway, and *k* denotes the number of genes of interest in that pathway.

### Identification of REO-based Tamoxifen-resistance Signature

Taking the consistent DEGs between tissues and cell lines as candidate genes, we used the Cox model and C-index analysis (Harrell et al., 1984) to develop a tamoxifen-resistance signature. The detailed process was described as follows.

#### Step 1: Selecting Survival-related Gene Pairs

(1) For the *n* candidate DEGs, pairwise comparisons were performed for all genes (generating a total of $C_{n}^{2}$ gene pairs), and this gene pair set was defined as Set 1. (2) From all gene pairs (*G*_*i*_, *G*_*j*_) in Set 1, the Cox model was used to select those that were significantly correlated with RFS of the tamoxifen-treated breast cancer patients. The set of significantly correlated gene pairs (FDR < 10%) was defined as Set 2.

#### Step 2: Optimizing the Gene Pair Signature

First, we enumerated all the gene pair combinations in Set 2. For each gene pair combination in a sample, if at least half of the gene pairs in the combination were consistent with tamoxifen sensitivity, the sample was identified as low risk; otherwise, it was considered high risk. Then, we calculated the C-index value for each gene pair combination, and selected the combination with maximum C-index as our tamoxifen-resistance signature (Set 3).

## Results

### Identification and Evaluation of DEGs in Cell Lines

**FIGURE 1:**
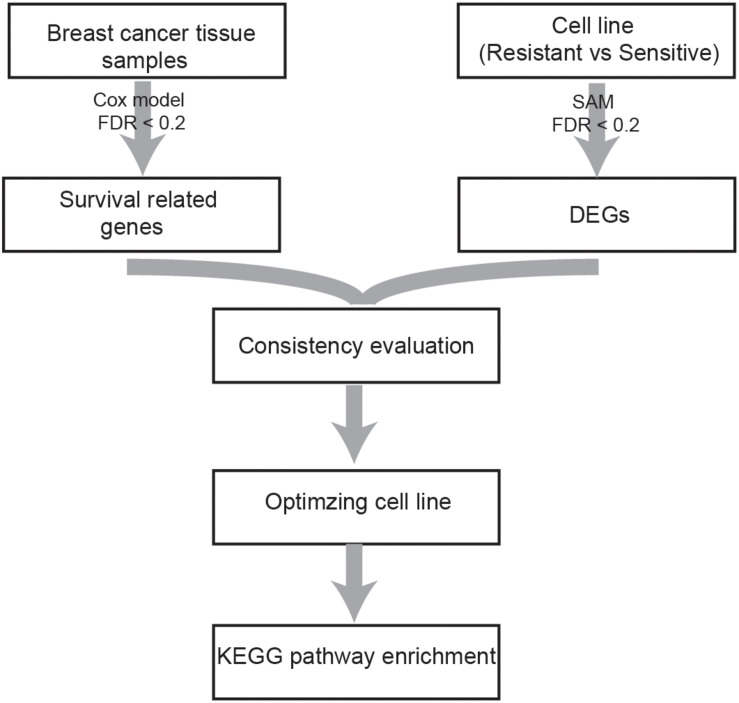
Flowchart of the analysis procedure.

A flowchart of the analysis procedure is shown in Figure 1. We identified the DEGs between tamoxifen-resistant and tamoxifen-sensitive cell line samples within each of the nine datasets using the SAM method (FDR < 20%). We also evaluated the consistency of DEGs among different datasets (a total of $C_{9}^{2}=36$ combinations). Among the 36 combinations, only 16 showed significant consistency (*p* < 0.05), as described in Table 2. These results indicate that there is greater heterogeneity among cell lines from different sources.

**TABLE 2:** Consistency evaluation of DEGs from different cell line datasets.

GEO Acc	Cell line*	Def_gene	Com_gene	Con_gene	Ratio	*P*
GSE27473	si-ER MCF7: MCF7	15937	10795	6147	0.5694	<1.00E-16
GSE14986	T8/17/29/52: MCF7	13391				
GSE27473	si-ER MCF7: MCF7	15937	12580	7427	0.5904	<1.00E-16
GSE21618	TamR: WT	15481				
GSE27473	si-ER MCF7: MCF7	15937	9675	5424	0.5606	<1.00E-16
GSE67916	MCF-7/TAMR:MCF-7	12227				
GSE27473	si-ER MCF7: MCF7	15937	8074	4450	0.5512	<1.00E-16
GSE118713	MCF-7/TAMR:MCF-7	10023				
GSE14986	T8/17/29/52: MCF7	13391	10494	7391	0.7043	<1.00E-16
GSE21618	TamR: WT	15481				
GSE14986	T8/17/29/52: MCF7	13391	8125	5396	0.6641	<1.00E-16
GSE67916	MCF-7/TAMR:MCF-7	12227				
GSE14986	T8/17/29/52: MCF7	13391	6534	4139	0.6335	<1.00E-16
GSE118713	MCF-7/TAMR:MCF-7	10023				
GSE14986	T8/17/29/52: MCF7	13391	6505	4042	0.6214	<1.00E-16
GSE125738	T47D-TR:T47D	10685				
GSE21618	TamR: WT	15481	9331	5386	0.5772	<1.00E-16
GSE67916	MCF-7/TAMR:MCF-7	12227				
GSE26459	G11OH-T: B7	6375	5525	3192	0.5777	<1.00E-16
GSE27473	si-ER MCF7: MCF7	15937				
GSE21618	TamR: WT	15481	7729	4189	0.5420	8.22E-14
GSE118713	MCF-7/TAMR:MCF-7	10023				
GSE118713	MCF-7/TAMR:MCF-7	10023	5808	3161	0.5442	8.16E-12
GSE125738	T47D-TR:T47D	10685				
GSE21618	TamR: WT	15481	7597	4061	0.5346	9.04E-10
GSE125738	T47D-TR:T47D	10685				
GSE67916	MCF-7/TAMR:MCF-7	12227	5824	3212	0.5515	2.00E-15
GSE118713	MCF-7/TAMR:MCF-7	10023				
GSE26459	G11OH-T: B7	6375	3767	2044	0.5426	9.10E-08
GSE118713	MCF-7/TAMR:MCF-7	10023				
GSE27473	si-ER MCF7: MCF7	15937	7991	4163	0.5210	9.32E-05
GSE125738	T47D-TR:T47D	10685				
GSE26459	G11OH-T: B7	6375	1163	521	0.4480	1.00E + 00
GSE12708	SUM44/LCCTam: SUM44	2538				
GSE26459	G11OH-T: B7	6375	52	21	0.4038	9.37E-01
GSE8562	MCF7/XBP1: MCF7	97				
GSE26459	G11OH-T: B7	6375	4623	2084	0.4508	1.00E + 00
GSE14986	T8/17/29/52: MCF7	13391				
GSE26459	G11OH-T: B7	6375	5262	2643	0.5023	3.76E-01
GSE21618	TamR: WT	15481				
GSE26459	G11OH-T: B7	6375	4090	1946	0.4758	9.99E-01
GSE67916	MCF-7/TAMR:MCF-7	12227				
GSE26459	G11OH-T: B7	6375	3750	1321	0.3523	1.00E + 00
GSE125738	T47D-TR:T47D	10685				
GSE27473	si-ER MCF7: MCF7	15937	2264	1056	0.4664	9.99E-01
GSE12708	SUM44/LCCTam: SUM44	2538				
GSE27473	si-ER MCF7: MCF7	15937	89	33	0.3708	9.95E-01
GSE8562	MCF7/XBP1: MCF7	97				
GSE12708	SUM44/LCCTam: SUM44	2538	23	12	0.5217	5.00E-01
GSE8562	MCF7/XBP1: MCF7	97				
GSE12708	SUM44/LCCTam: SUM44	2538	1885	702	0.3724	1.00E + 00
GSE14986	T8/17/29/52: MCF7	13391				
GSE12708	SUM44/LCCTam: SUM44	2538	2134	920	0.4311	1.00E + 00
GSE21618	TamR: WT	15481				
GSE12708	SUM44/LCCTam: SUM44	2538	1676	862	0.5143	1.25E-01
GSE67916	MCF-7/TAMR:MCF-7	12227				
GSE12708	SUM44/LCCTam: SUM44	2538	1588	625	0.3936	1.00E + 00
GSE118713	MCF-7/TAMR:MCF-7	10023				
GSE12708	SUM44/LCCTam: SUM44	2538	1630	840	0.5153	1.12E-01
GSE125738	T47D-TR:T47D	10685				
GSE8562	MCF7/XBP1: MCF7	97	80	42	0.5250	3.69E-01
GSE14986	T8/17/29/52: MCF7	13391				
GSE8562	MCF7/XBP1: MCF7	97	84	46	0.5476	2.23E-01
GSE21618	TamR: WT	15481				
GSE8562	MCF7/XBP1: MCF7	97	57	30	0.5263	3.96E-01
GSE67916	MCF-7/TAMR:MCF-7	12227				
GSE8562	MCF7/XBP1: MCF7	97	63	25	0.3968	9.62E-01
GSE118713	MCF-7/TAMR:MCF-7	10023				
GSE8562	MCF7/XBP1: MCF7	97	63	25	0.3968	9.62E-01
GSE125738	T47D-TR:T47D	10685				
GSE67916	MCF-7/TAMR:MCF-7	12227	5751	2910	0.5060	1.85E-01
GSE125738	T47D-TR:T47D	10685				

*Resistant and sensitive cell line samples from the corresponding dataset. Taking dataset GSE14986 as an example, among T8/17/29/52: MCF7, T8/17/29/52 denote resistant cell lines, MCF7 denotes sensitive cell line; Def_gene denotes the number of DEGs in the corresponding dataset; Com_gene denotes the number of overlapped DEGs between two datasets; Con_gene denotes the number of overlapping DEGs with the same dysregulation between two datasets; Ratio denotes the consistency ratio of DEGs.

### Identification of Tamoxifen Survival-related Genes in Tissues

Based on the univariate Cox regression model with FDR < 20%, 893 and 968 tamoxifen survival-related genes were identified in datasets GSE17705 and GSE6532, respectively; 235 genes were common to the two groups, all of which had the same dysregulation direction (which could not occur by chance; binomial test, *p* < 1.0E-16), further verifying the reliability of the results. These 235 genes were considered to be breast cancer tissue candidate genes.

Owing to the heterogeneity among cell lines, we evaluated the consistency between tissue candidate genes and DEGs from different cell line datasets (resistant vs sensitive) to select an optimal cell line model that could well represent the characteristics of clinical tissue samples. We found that only the DEGs from dataset GSE26459 were well reproduced among tissue candidate genes; the consistency ratio was above 73%, indicating that this did not occur by chance (binomial test, *p* = 2.13E-07). The DEGs from the other cell line datasets were not well reproduced among the tissue candidate genes (Table 3). These results demonstrate that the cell line data from dataset GSE26459 could well represent the characteristics of clinical breast cancer tissue samples.

**TABLE 3:** Consistency evaluation between tissues and cell lines.

GEO Acc	Def_gene	Com_gene	Con_gene	Ratio	*P*
GSE26459	6375	114	84	0.7368	2.13E-07
GSE27473	15937	211	93	0.4408	9.63E-01
GSE12708	2538	46	15	0.3261	9.94E-01
GSE8562	97	5	3	0.6000	5.00E-01
GSE14986	13391	178	55	0.3090	1.00E + 00
GSE21618	15481	207	82	0.3961	9.99E-01
GSE67916	12227	162	61	0.3765	9.99E-01
GSE118713	10023	159	63	0.3962	9.97E-01
GSE125738	10685	159	32	0.2013	1.00E + 00

*Def_gene denotes the number of DEGs in the corresponding dataset; Com_gene denotes the number of overlapping DEGs between the 235 tissue candidate genes and the corresponding cell line dataset; Con_gene denotes the number of overlapping DEGs with the same dysregulation between two datasets; Ratio denotes the consistency ratio of DEGs.*

### KEGG Pathway Enrichment

KEGG pathway enrichment analysis was performed for the 235 tissue candidate genes from datasets GSE17705 and GSE6532 using a threshold of FDR < 0.2, and for the DEGs from cell line dataset GSE26459 using the same threshold (Table 4). There was no pathway commonly enriched between tissues and the cell line, possibly owing to the low statistical power (Zou et al., 2011) or to partial differences between resistant and sensitive cell lines induced by tamoxifen treatment (Dancik et al., 2011). Thus, taking the pathways enriched in tissues as the gold standard, we obtained the *p*-values of these pathways in dataset GSE26459 (Table 4). With *p* < 0.2, the cell cycle, p53 signaling pathway, oocyte meiosis, and progesterone-mediated oocyte maturation were recurring themes in the pathway analysis for both tissues and cell lines. These pathways have been reported to be correlated with tamoxifen resistance.

**TABLE 4:** KEGG pathway enrichment of tissue and cell line.

Tissue			Cell line		
	
Pathway num	Pathway name^a^	P*	Pathway num	Pathway name^b^	FDR
hsa04110	Cell cycle	0.0270	hsa03013	RNA transport	4.62E-08
hsa04115	p53 signaling pathway	0.0226	hsa03010	Ribosome	1.14E-05
hsa04114	Oocyte meiosis	0.0726	hsa00970	Aminoacyl-tRNA biosynthesis	1.82E-05
hsa04914	Progesterone-mediated oocyte maturation	0.1176	hsa03008	Ribosome biogenesis in eukaryotes	1.64E-04
hsa03440	Homologous recombination	0.3907	hsa03040	Spliceosome	7.40E-04
hsa04672	Intestinal immune network for IgA production	0.8288	hsa03410	Base excision repair	1.98E-03
hsa04060	Cytokine-cytokine receptor interaction	0.9977	hsa00620	Pyruvate metabolism	9.57E-03
			hsa01230	Biosynthesis of amino acids	0.0119
			hsa01100	Metabolic pathways	0.0194
			hsa01212	Fatty acid metabolism	0.0194
			hsa01200	Carbon metabolism	0.0214
			hsa00510	N-Glycan biosynthesis	0.0244
			hsa00531	Glycosaminoglycan degradation	0.0244
			hsa04360	Axon guidance	0.0244
			hsa04612	Antigen processing and presentation	0.0244
			hsa04917	Prolactin signaling pathway	0.0257
			hsa00511	Other glycan degradation	0.0272
			hsa04144	Endocytosis	0.0272
			hsa03018	RNA degradation	0.0300
			hsa04142	Lysosome	0.0322
			hsa04330	Notch signaling pathway	0.0513
			hsa01040	Biosynthesis of unsaturated fatty acids	0.0573
			hsa04722	Neurotrophin signaling pathway	0.0754
			hsa04910	Insulin signaling pathway	0.0872
			hsa01210	2-Oxocarboxylic acid metabolism	0.0945
			hsa04141	Protein processing in endoplasmic reticulum	0.1101
			hsa00280	Valine, leucine and isoleucine degradation	0.1121
			hsa04120	Ubiquitin mediated proteolysis	0.1121
			hsa00270	Cysteine and methionine metabolism	0.1319
			hsa00020	Citrate cycle (TCA cycle)	0.1527
			hsa03050	Proteasome	0.1848

*Tissue: ^*a*^KEGG pathway enriched for survival-related genes in tissues (FDR < 0.2); *P* denotes the *p*-value for a KEGG pathway, enriched for tissues, in the cell line dataset GSE26459. Cell line:^*b*^KEGG pathway enriched by DEGs between resistant and sensitive cell lines in dataset GSE26459 (FDR < 0.2).*

Studies have shown that tamoxifen could affect the cell cycle of human breast cancer cell lines, the major sensitivity to tamoxifen in terms of both inhibition of cell cycle progression and drug cytotoxicity occurring particularly in the G0-G1 stage (Taylor et al., 1983). Tamoxifen could also affect the mitosis of oocytes and lead to premature centromere separation (London and Mailhes, 2001). The ***PTEN*** protein, encoded by the gene, in the p53 signaling pathway has been shown to be associated with tamoxifen resistance (Shoman et al., 2005). Similarly, the ***PGR*** protein in the progesterone-mediated oocyte maturation signaling pathway has been shown to be associated with tamoxifen response (Elledge et al., 2000). In summary, the pathways found to be enriched in tissues and also in cell line dataset GSE26459 (*p* < 0.2) were correlated with tamoxifen resistance, further demonstrating that the cell line model from dataset GSE26459 could represent the characteristics of clinical tissue samples.

Moreover, with FDR < 20%, the DEGs from cell line dataset GSE26459 were enriched in 31 pathways, compared with only seven pathways for the genes from tissue samples. However, as shown in Table 4, many of the pathways enriched for the cell lines from dataset GSE26459 are associated with tamoxifen treatment. For example, the prolactin signaling pathway and neurotrophin signaling pathway are related to side effects of tamoxifen (Lamberts et al., 1982; El-Ashmawy and Khalil, 2014), indicating that some of the differences between resistant and sensitive cell lines were due to tamoxifen treatment.

### Identification of Tamoxifen Response Signature

**FIGURE 2:**
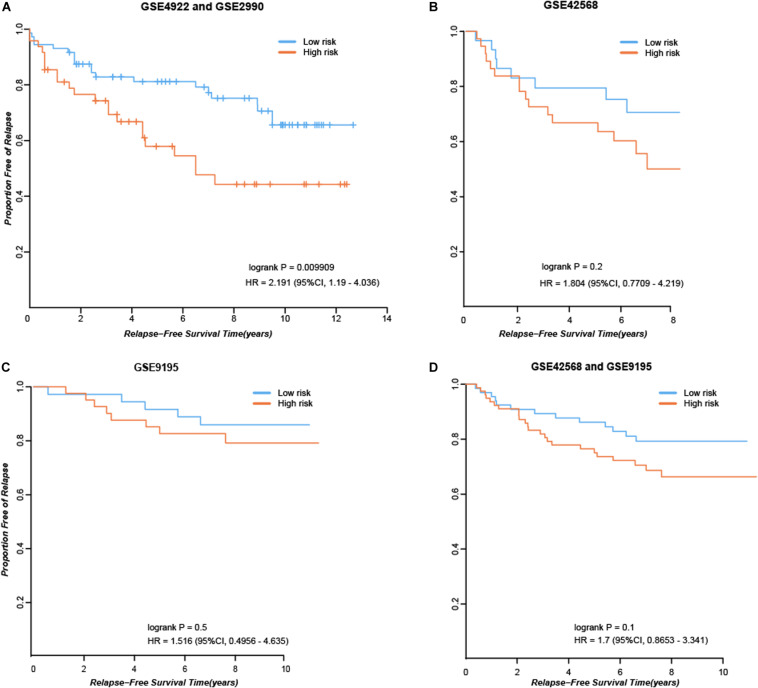
Performance of our signature in independent dataset. **(A)** RFS curves in the combined data from datasets GSE4922 and GSE2990. **(B)** RFS curves in the dataset GSE42568. **(C)** RFS curves in the dataset GSE9195. **(D)** RFS curves in the combined data from datasets GSE42568 and GSE9195.

First, we considered the 84 consistent DEGs between tissues and cell line dataset GSE26459 to be clinical tamoxifen-resistance-related genes. In the training dataset GSE12093, pairwise comparisons were performed for all clinical tamoxifen-resistance-related genes, and all the gene pairs were analyzed with a univariate Cox regression model. With FDR < 10%, 20 gene pairs were identified that were significantly associated with RFS. Then, among the 20 gene pairs, we enumerated all the gene pair combinations to calculate their C-index values, and selected the gene combination with the maximum C-index as the tamoxifen response signature. Finally, two gene pairs (***TOP2A***, ***SLC7A5***; ***NMU***, ***PDSS1***) were identified. Based on our signature and the majority vote rule, the training dataset samples could be divided into high- and low-risk samples, which had significantly different RFS (hazard ratio [HR] = 9.509, logrank *p* = 1.98E-07). Our signature was also verified in an independent validation test using combined data from datasets GSE4922 and GSE2990 (HR = 2.191, logrank *p* = 0.009909), as shown in Figure 2A. Moreover, we searched public databases again for breast cancer tissue samples treated only with post-operative tamoxifen, for which associated RFS information was available, to further verify the performance of our signature. Finally, two new independent datasets were obtained. For the breast cancer tissue samples from dataset GSE42568, 37 samples were identified as high risk, and 30 were identified as low risk (HR = 1.804, logrank *p* = 0.2), as shown in Figure 2B. For the breast cancer tissue samples from dataset GSE9195, 41 samples were identified as high risk and 36 as low risk (HR = 1.516, logrank *p* = 0.5), as shown in Figure 2C. Although the difference between the groups was not significant according to statistical tests, there was a clear trend indicating a difference in RFS between the high- and low-risk groups identified by our signature (Figure 2B-C). Moreover, we combined the above two datasets to further verify the performance of our signature. In the combined data from datasets GSE42568 and GSE9195, 78 samples were identified as high risk and 66 samples were identified as low risk (HR = 1.7, logrank *p* = 0.1), as shown in Figure 2D. In summary, the results indicate that our signature (consisting of two gene pairs) can predict drug efficacy to some extent.

## Discussion

Cell line models are widely used in various fields of medical research, especially in basic cancer research and drug discovery (Masters, 2000; Mirabelli et al., 2019). Despite the successful application of cell lines in basic research, their use as model systems remains controversial (Masters, 2002; Sandberg and Ernberg, 2005; Peng et al., 2018; Hallas-Potts et al., 2019). Owing to issues such as cross-contamination, mislabeling, or the identification of drug resistance, some cell line models do not adequately represent the characteristics of clinical tissues. In this study, based on evaluation of the consistency of DEGs between tissues and cell lines, we selected the optimal cell line model to represent the characteristics of clinical tissue samples; this was further verified by pathway analysis. Our analysis method is also suitable for other types of cell line modes.

The tamoxifen survival-related genes identified in tissue samples from different datasets were significantly consistent, suggesting that the results were reliable. However, the DEGs found in tamoxifen-resistant and tamoxifen-sensitive cell lines from different sources were less reproducible, indicating that cell line models from different sources show more heterogeneity. Therefore, it will be of great clinical significance to screen for drug-resistant and drug-sensitive cell line models that better represent the characteristics of clinical tissue samples. According to our results, the DEGs from cell line dataset GSE26459 were reproducible in tissue samples, indicating that the cell line model from this dataset was representative of the characteristics of clinical tissue samples. Tissue samples were obtained by surgical resection before tamoxifen therapy. Thus, the survival-related genes obtained from tissues were intrinsic to the patient and not induced by tamoxifen treatment. The resistant and sensitive cell lines from dataset GSE26459 were selected from MCF subclones (Gonzalez-Malerva et al., 2011); this might partly explain why the cell lines from GSE26459 could represent the characteristics of clinical tissue samples. The pathways enriched in tissues and in cell line dataset GSE26459 (*p* < 0.2) have been reported to be associated with tamoxifen resistance (Lamberts et al., 1982; El-Ashmawy and Khalil, 2014). Moreover, the clinical tamoxifen-resistance gene-pair signature we developed was verified in independent validation dataset, which indicates that our signature has some power to predict response to tamoxifen therapy, and further demonstrates that we have selected appropriate tamoxifen-resistant and tamoxifen-sensitive cell line models.

Although the cell line models identified by our analytical method could well reflect the information of clinical tissue samples, there were some limitations. As patients with breast cancer usually have good prognosis, the endpoint of their follow-up is usually survival or recurrence time. Furthermore, as well as the effects of drugs, many factors including mood, marital status, and economic status could affect the survival of patients. The above factors might cause that some of the survival-related genes that we have identified are not involved in tamoxifen resistance. In future work, use of more tissue sample data or an improved algorithm should be considered. Moreover, as DNA methylation patterns, genomic changes, etc., might also predict sensitivity to drugs, the use of other types of data (such as microRNAs, DNA methylations, and genomic changes) in cell line model optimization deserve consideration in future studies.

## Data Availability Statement

All datasets presented in this study are included in the article/supplementary material.

## Author Contributions

QZG and XKS conceived the study, analyzed the data, produced the figures, performed the statistical analysis, and drafted the manuscript. ZZZ participated in the revision of the manuscript. YZZ and YTC searched the data and participated in the statistical analysis. JL conceived the study and participated in its design and coordination, helped to draft the manuscript, and supervised the work. All authors contributed to the article and approved the submitted version.

## Conflict of Interest

The authors declare that the research was conducted in the absence of any commercial or financial relationships that could be construed as a potential conflict of interest.

## Abbreviations

DEGs: differentially expressed genes
GEO: Gene Expression Omnibus
ER +: estrogen receptor positive
KEGG: Kyoto Encyclopedia of Genes and Genomes
REO: relative expression ordering
RFS: relapse-free survival
SAM: significance analysis of microarrays.
